# Extract from the Zooxanthellate Jellyfish *Cotylorhiza tuberculata* Modulates Gap Junction Intercellular Communication in Human Cell Cultures

**DOI:** 10.3390/md11051728

**Published:** 2013-05-22

**Authors:** Antonella Leone, Raffaella Marina Lecci, Miriana Durante, Stefano Piraino

**Affiliations:** 1 Institute of Sciences of Food Production, National Research Council, Unit of Lecce (CNR, ISPA), Via Prov.le Lecce-Monteroni, Lecce 73100, Italy; E-Mails: raffaella.lecci@ispa.cnr.it (R.M.L.); miriana.durante@ispa.cnr.it (M.D.); 2 CoNISMa, National Interuniversity Consortium on Marine Sciences, Local Unit of Lecce, Via Prov.le Lecce-Monteroni, Lecce 73100, Italy; E-Mail: stefano.piraino@unisalento.it; 3 University of Salento, Via Prov.le Lecce-Monteroni, Lecce 73100, Italy

**Keywords:** bioactive compounds, jellyfish extracts, zooxanthellae, gap junction intercellular communications, anticancer and antioxidant activity

## Abstract

On a global scale, jellyfish populations in coastal marine ecosystems exhibit increasing trends of abundance. High-density outbreaks may directly or indirectly affect human economical and recreational activities, as well as public health. As the interest in biology of marine jellyfish grows, a number of jellyfish metabolites with healthy potential, such as anticancer or antioxidant activities, is increasingly reported. In this study, the Mediterranean “fried egg jellyfish” *Cotylorhiza tuberculata* (Macri, 1778) has been targeted in the search forputative valuable bioactive compounds. A medusa extract was obtained, fractionated, characterized by HPLC, GC-MS and SDS-PAGE and assayed for its biological activity on breast cancer cells (MCF-7) and human epidermal keratinocytes (HEKa). The composition of the jellyfish extract included photosynthetic pigments, valuable ω-3 and ω-6 fatty acids, and polypeptides derived either from jellyfish tissues and their algal symbionts. Extract fractions showed antioxidant activity and the ability to affect cell viability and intercellular communication mediated by gap junctions (GJIC) differentially in MCF-7and HEKa cells. A significantly higher cytotoxicity and GJIC enhancement in MCF-7 compared to HEKa cells was recorded. A putative action mechanism for the anticancer bioactivity through the modulation of GJIC has been hypothesized and its nutraceutical and pharmaceutical potential was discussed.

## 1. Introduction

The marine environment has been recognized as a rich source of bioactive metabolites with various biological and pharmacological activities. The chemical complexity and biological diversity of the marine-derived compounds is enormous, so that bioprospecting of marine organisms represents today a major tool for the discovery of new therapeutic agents and drug candidates [1,2,3,4,5,6,7,8].

Cnidarians polyps and jellyfish have increasingly become an attractive source of physiologically active compounds. Extracts from different species were reported to exert hemolytic [9], insecticidal [10], cardiovascular [11], antioxidant [12], anti-microbial [13], and cytotoxic [9] effects. Strong fibrinogenolytic factors have been recently found in the moon jellyfish *Aurelia aurita* tentacle extract [14]. Partially purified venom from the mauve stinger jellyfish *Pelagia noctiluca* displayed potent anti-tumoral properties against U87 cells [15]. Collagen hydrolysate from edible jellyfish *Rhopilema esculentum* exerted antioxidant and protective effects on mice skin subjected to photo aging induced by UV irradiation [16]. Collagen from giant jellyfish *Nemopilema nomurai* has been shown to exert immunostimulatory effect on hybridoma cell line HB4C5 and human peripheral blood lymphocytes [17,18].

Marine jellyfish are currently recognized as subject to worldwide proliferations in coastal areas, becoming a crucial ecological and societal issue in recent decades [19]. Occurrences of jellyfish outbreaks increasingly interfere with human economic and recreational activities, such as bathing, fishery, tourism, *etc.*, as well as the public health [20] and coincide with human proliferations and man-induced environmental perturbations [21]. Jellyfish have been reported to clog fishing nets, spoil commercial catches, cause serious damage to aquaculture, clog the cooling systems of coastal power plants, and sting or even kill tourist swimmers [22,23,24,25,26,27,28,29]. Conversely, under an opposite perspective, the large amount of jellyfish biomass could be considered as a valuable source of bioactive compounds including bioactive peptides, collagen and gelatin, oligosaccharides, fatty acids, enzymes, calcium, water-soluble minerals, and biopolymers. The identified various biological activities, including antioxidant activity, make them a potentially valuable material for food, cosmetic, and biomedical industries, such as has been proposed for seafood processing by-products [30].

The scyphozoan *Cotylorhiza tuberculata* (Macrì, 1778) [31], the most common rhizostome jellyfish in the Mediterranean Sea, is subject to summer population outbreaks. High jellyfish abundances can be found in enclosed bays such as Vlyho Bay in the Ionian Island of Lefkada-Greece [32] and in coastal lagoons, such as the Mar Menor in the western Mediterranean Sea where annual blooms have been observed since 1995 [33]. To protect human leisure activities from high *C. tuberculata* abundance in Mar Menor, local maritime authorities now constantly implement management plans by use of fishing vessels for removal of large jellyfish biomasses or by use of protective coastal nets to create safe bathing areas [34]. In 2002 and 2003, approximately five thousands tons of *C. tuberculata* were yearly removed during summers months [35].

An important biological aspect, linked to the success of *C. tuberculata* populations, is the occurrence of endosymbiotic, unicellular dinoflagellates, usually referred as zooxanthellae. As for several other rhizostome jellyfish species and other cnidarians [32,36,37], *C. tuberculata* hosts in its endodermal tissue a dense population of the dinoflagellate *Symbiodinium* (=*Gymnodinum*) *microadriaticum* (Freudenthal) [38]. The endosymbiotic association occurs very early in the life cycle of *C. tuberculata* [37] and the occurrence of zooxanthellae in the polyp stage is required to activate the process of medusa formation (strobilation) of *C. tuberculata* [32,37,39]. Apparently, the importance of autotrophy provided by zooxanthellae seems to be lower than heterotrophic uptakes for jellyfish growth and survival [39,40]. Nevertheless, we observed that unfed polyps maintained for two years under natural daylight conditions and laboratory room temperature in closed aquarium system, did not show any sign of ageing or reduction in size. The molecular regulation and maintenance of the symbiotic relationships between the microalgae and their animal hosts is still largely unknown, but algal-derived metabolites, resulting from photosynthetic framework and secondary metabolism play crucial roles in homeostatic mechanisms [41,42]. In *Symbiodinium* dinoflagellates, the majority of the photosynthetic pigments are associated to light-harvesting pigment-protein complexes [43,44,45]. These photosynthetic pigments are believed to be central to photoprotection in jellyfish-associated *Symbiodinium*, by stabilization of the chloroplast thylakoids, and vital to the prevention or quenching of ROS in the host [45,46]. Another key area of metabolic activity within the symbiosis is the free fatty acid synthesis and translocation within the dinoflagellate symbionts and their cnidarian host. Indeed, lipids are the main energy stores in cnidarians and the primary products derived from photosynthetically fixed carbon and translocated from the dinoflagellate symbionts to the host [47]. Total lipids from the symbiosis (triacylglycerols, wax esters, phospholipids and free fatty acids), account for 10%–46% of the cnidarian tissue dry weight [48,49]. Production and accumulation of long-chain, highly unsaturated fatty acids (HUFA), *n*-3 and *n*-6, such as DHA [22:6 (*n*-6)], is well documented [49,50]. Beyond the significance of essential FAs in symbiosis regulation, the importance of *n*-3 and *n*-6 derived essential FAs, such as DHA, to supplement animal diets is well known [51,52]. Essential fatty acids (EFA) are PUFAs involved in biological processes, but not synthesized by animal cells. These fatty acids represent a necessary component of animal diets, since they are used as starting points for building longer chains of fatty acids and their deficiency may lead to severe damage to the organism. These FAs are produced by the symbiotic microalgae and, in some cases, in large amounts [53]. Therefore, the relationship with the symbiotic dinoflagellate *S. microadriaticum* makes the jellyfish *C. tuberculata* a heterogeneous and complex biomass consortium rich in diverse and potentially bioactive compounds.

The chemical, biological and ecological diversity of marine metabolites has largely contributed to the discovery of potent compounds with strong antitumor activities [54]. However, the structural diversity of the marine compounds, from simple linear peptides to complex macrocyclic polyethers, represents one of the first difficulties in new drug discovery from marine natural products. Recent advances in sophisticated technologies for the isolation and characterization of marine natural products and the development of high-throughput screening methods have substantially increased the rate of discovery of various compounds of biomedical application. High-throughput screening, combinatorial chemistry and, most recently, *in silico* virtual screening techniques partly supported successful attempts to identify new drug candidates. However, the classical approach to recognize bioactive compounds through analysis of their specific biologic activity remains highly effective, especially when associated to the above-mentioned technological approaches.

One of the mechanisms underlying the multistage carcinogenesis process is the homeostatic regulation disorder related to abnormal gap junction intercellular communications (GJIC).

GJ are communicating junctions that are present in most cells in animal tissue. The GJ channels are formed by four-pass transmembrane proteins sharing a similar function and overall structure but encoded from different gene families, pannexins and connexins in chordates, and pannexins and innexins in invertebrates. The molecular architecture of GJIC consists of a couple of hexameric structures in the membrane of two adjacent cells, docking each other and forming a channel with a central pore, which allows the diffusion of metabolites, ions, small signaling molecules, and mediate electrical synapses [55,56,57]. Gap junctions are grouped in plaques at the cell plasma membrane surface allowing molecules smaller than about 1–2 kDa to pass directly from the cytoplasm of one cell to the cytoplasm of another adjacent cell. Traditionally, the function of GJ proteins was associated to the formation of membrane channels; however, recent studies revealed additional functions, including sensing of the extracellular environment, cell-cell adhesion, facilitation of cell migration, and modulation of endocrine, pain, signal transduction and apoptotic pathways [58,59].

The mechanisms controlling GJ functionality are strictly regulated [60] at multiple levels, ranging from gene transcription to gap junction trafficking and degradation [59]. Connexin phosphorylation is involved both in changing single channel conductance and in protein trafficking to the cell surface and degradation. The regulation of GJIC by connexin phosphorylation is quite complex, as the outcome of this post translational modification is both connexin- and kinase specific [61,62,63,64].

GJIC has been speculated to be a necessary, if not sufficient, biological function of metazoan cells for the regulation of growth control, differentiation and apoptosis of normal progenitor cells, able to regulate both tissue homeostasis and the triggering of intra-cellular signal transduction mechanisms. Normal, contact-inhibited cells have functional GJIC, while most, if not all, tumor cells have dysfunctional homologous or heterologous GJIC [65]. Cancer cells are characterized by the lack of growth control, inability to terminally differentiate or apoptose under normal conditions and have extended or immortalized life spans. Cancer cells either have no connexin expression or have expressed connexins but no functional GJIC; as a consequence, cancer cells lose the ability to respond appropriately to extra-cellular stimuli [66]. In this context it would seem that GJIC is the ultimate down-stream cell function that must be maintained to prevent cancer [67]. The classical model of carcinogenesis begins with an *initiation* step, when the exposure to a carcinogen results in an irreversible genetic change within a single cell. The reversible down regulation of GJIC plays a role during the *promotion* phase of carcinogenesis and the presence of GJIC is closely linked to the suppression of tumorigenic phenotypes [65,68]. In normal tissues, GJIC is necessary for apoptosis and by blocking apoptosis with chemicals, it is possible to promote initiated premalignant cells or by increasing apoptosis by increasing GJIC, one could prevent tumorigenesis [68]. Increasing evidences indicated that prevention of GJIC during the *promotion* phase or the up-regulation of GJIC in tumorigenic cells represents a mechanistic-based strategy for chemoprevention or chemotherapy [69,70]. A consistent observation is that many chemopreventive compounds such as phytochemicals [70,71], antitumor-promoting agents and anticancer drugs can reverse the down-regulation of GJIC [65,68,72,73,74,75,76].

As the inhibition of GJIC is considered an *in vitro* biomarker of tumor promotion, the GJIC enhancement, through several processes, could be regarded as anti-tumor mechanism. The GJIC modulation can be proposed as an antitumor mechanism underlying the action of several natural bioactive compounds [70].

Several bioactive marine compounds may affect GJIC. For instance, the depsipeptides isolated from the sponge *Geodia corticostylifera* have antiproliferative activity on breast cancer cells and increases the size of GJ plaques in HTC-Cx43-GFP cells [77]. Astaxanthin and canthaxanthin, xanthophylls synthesized by microalgae and yeast, with demonstrated cancer preventive effect in animal studies, were able to modulate GJIC, inducing changes in the phosphorylation state of connexin 43 proteins [78,79].

Conversely, similarly to several tumor promoter chemicals, the potential tumor promoting effects of several marine cyanobacteria seems related to their ability to inhibit GJIC [80].

From a computational analysis of naturally occurring marine compounds, based on protein-ligand interactions, many compounds have shown selective interaction towards gap junction and cell adhesive communication proteins resulting as ligand against the specific target molecules [81]. Despite the limits of this approach, many molecules reported for other interesting bioactivities, such as the anti-inflammatory sesterterpene manoalide [82,83,84] were identified as good ligands of the GJ connexin 43 and connexin 26 proteins. Dihydroxrytetrahydrofurans, some stereoisomers of which had been recognized as metabolites of the marine brown alga *Notheia anomala* [85], ascosalipyrrolidinone A [86], an antimicrobial alkaloid isolated from the endophytic obligate marine fungus *Ascochyta salicorniae*, and solenolide A, a briariane diterpene lactone from the cnidarian octocoral *Solenopodium excavatum* [87,88], they all resulted as ligands specifically against GJ protein targets, connexin 43 and connexin 26 [81]. 

The present study aimed to isolate and identify potentially bioactive compound(s) occurring in the tissues of *C. tuberculata* jellyfish. Here the extraction, fractioning and biological activity assessment of different fractions of a jellyfish hydroalcoholic extract are described. The effects of the extract fractions on cancer (MCF-7) and non cancer (HEKa) cells were evaluated by cell-based assays allowing the quantification of cytotoxicity of jellyfish-derived compounds and their effect on the GJIC functionality as target of anti-tumor promoting activity. 

## 2. Results and Discussion

### 2.1. Jellyfish Sample Features

**Figure 1:**
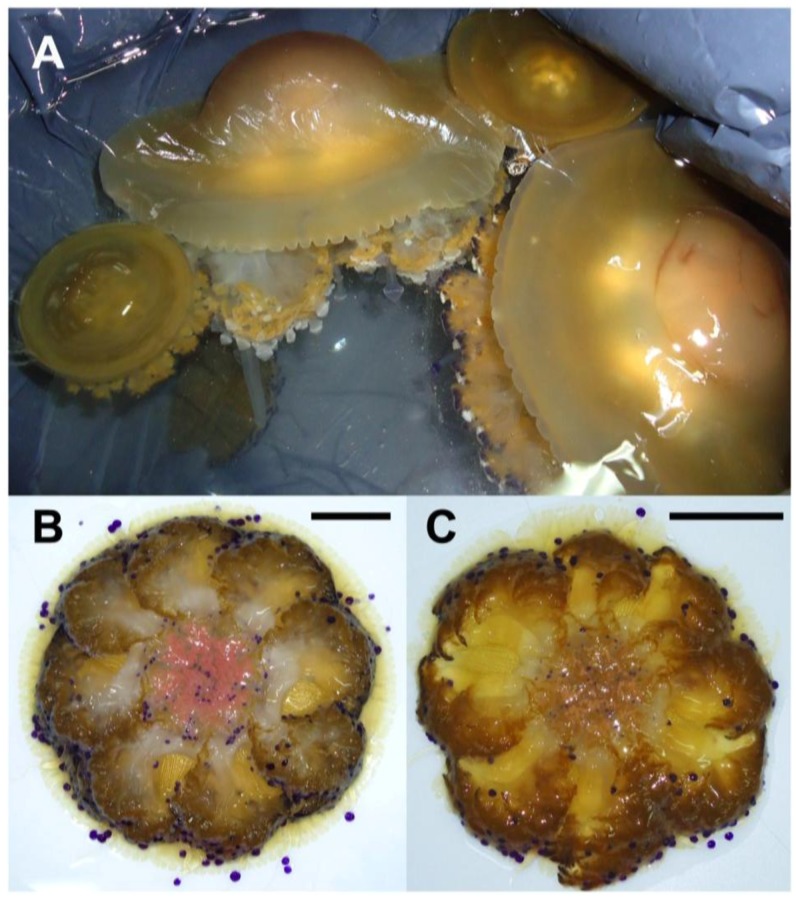
(**A**) *C. tuberculata* specimens of different size, collected in late summer; (**B**) Female jellyfish brood-carrying filaments and oral arm grooves (pink colored); (**C**) Male jellyfish with spermatic channels filled up of gametes and colored in light brown or white. Bar = 5 cm.

*Cotylorhiza* *tuberculata*, collected in late summer off the Apulia coasts, showed high variability in size (Figure 1, Table S1). The specimens ranged from about 6 cm to nearly 30 cm in bell diameter; the oral arms and tentacles usually expand beyond the increase in size of the umbrella. The mature organisms (with gametes or larvae), reached considerable size and mass, as showed from the weight/diameter ratios, which ranged from about 3 to 61 in younger and mature individuals, respectively (Table S1). 

Preliminary biometric analyses, limited to the summer 2011, showed that dry matter from *C. tuberculata* biomass, obtained by lyophilization, was increasing with the jellyfish size and represented until 30% of its fresh weight in mature organisms (Table S1). These data clearly indicated that the *Cotylorhiza* jellyfish biomass could be quantitatively significant and a considerable amount of material can be provided when jellyfish blooms occur.

**Figure 2:**
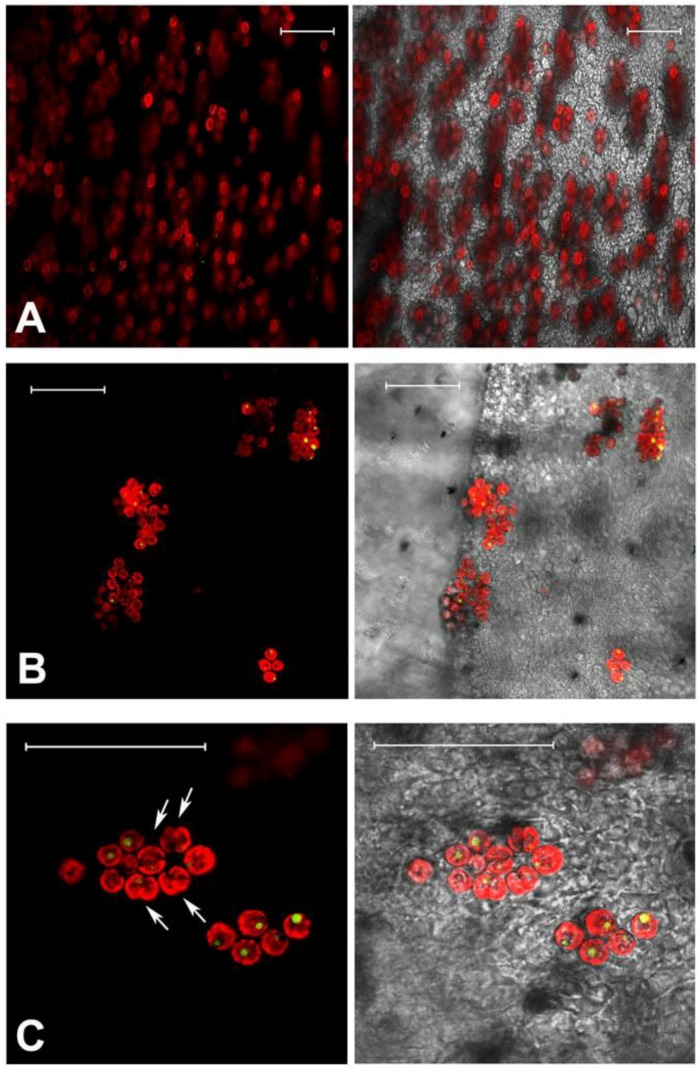
Confocal microscopy images of *C. tuberculata* tissues showing auto-fluorescence from zooxanthellae. Tissue samples from the frilled edges of oral arms (**A**); and umbrella (**B**); Magnification showing peripheral red auto-fluorescence of the chloroplast, green-fluorescence in the single vacuole and several dividing cells (*doublet*, arrowed) (**C**). Images are the merge of false-colored red and green autofluorescence emission (on the left), and the merge of red, green fluorescence and transmitted light (or bright field) picture (on the right). Bars = 50 μm.

The endosymbiotic algae *S. microadriaticum* is known to live in association within *C. tuberculata* in the Mediterranean Sea. The dinoflagellates are typically located in the jellyfish endodermal cells within symbiosomes: A perisymbiotic membrane, derived from the host cell plasmalemma, keeps the coccoid stage of the dinoflagellate separated from the jellyfish cell cytoplasm [89]. The color of the umbrella top and the frilled edges of the oral arms, appeared greenish-brown in the living tissue where high density of zooxanthellae in the mesoglea was observed (Figure 1, Figure 2A,B), mostly aggregated in small clusters and showing high division rates (Figure 2C). *Symbiodinum* spp. was present as both coccoid and motile biflagellate cell stages. However, most of the cells were in the coccoid stage, spherical, with homogeneous size of 8.4 ± 1.0 μm in diameter, with a single, peripheral, reticulated chloroplast, occupying a large part of the cell volume. Coccoid *Symbiodinium* cells appeared metabolically active, since they undergo mitosis (Figure 2C); therefore, high photosynthetic activity and product accumulation it is expected at this stage [90]. It was also evident a relatively large accumulation body (3.0 ± 0.5 μm in diameter), as a round shaped, reddish-brown vacuole, which displayed an unspecific green auto-fluorescence under confocal microscope, with emission peak between 505 and 530 nm (Figure 2C), similarly to previous observationsin *Symbiodinium*-coral symbiotic associations [91].

The green auto-fluorescence in the accumulation body resulted clearly separated from the red auto-fluorescence of chlorophylls and other photosynthetic pigments related to the chloroplast organelle, since the confocal microscopy allows for the resolution of multiple distinct fluorescent emissions associated with a single cell. Although a number of compounds are responsible for green-autofluorescence in vegetal organisms, green auto-fluorescence in algae is not well investigated, and the composition of the described fluorescing substances in different algal species is largely unknown. Recently, Tang and Dobbs [92] reported a green auto-fluorescence, which occurred in species of different algal classes (mainly dinoflagellates and diatoms) that interfered with detection of green-fluorescing stains. The reported connection [92,93] between the decrease of chlorophyll red auto-fluorescence, due to photodegradation, and the increase of “green autofluorescence” in algae, is not supported from our observations by confocal microscope, where the red-autofluorescence of chlorophylls in the chloroplast was not related to the green-autofluorescence in the accumulation body. Although the accumulation body content and its metabolic significance are still unknown, a role of storage of secondary metabolites, with potential biological activity, can be hypothesized. 

### 2.2. Hydro-Alcoholic Extraction and Fractioning

A hydroalcoholic extraction was performed from lyophilized samples of different *C. tuberculata* specimens. The extraction in 80% ethanol was used as mild extraction method suited to remove polar or mildly non-polar low molecular weight substances, such as small proteins, soluble carbohydrates and secondary metabolites. Unlike the variability of the fresh biomasses and the related amount of dry material, quite constant percentages of hydro-alcoholic extractable material was obtained (Tables S1 and S2). A considerable amount of extract was obtained from the lyophilized total tissue and its dry weight amounted to an average of 11.7 ± 1.7% of the fresh biomass and of 43.6 ± 4.1% of dry biomass (Table S2).

**Figure 3:**
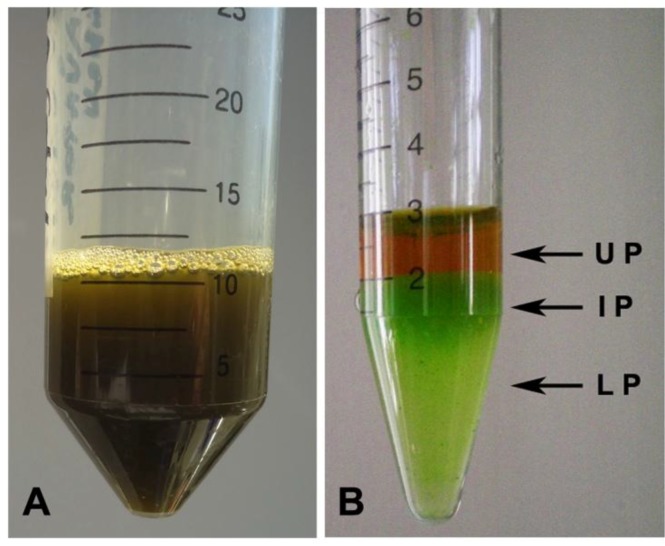
Ethanol extract from *C. tuberculata* tissues before (**A**) and after (**B**) acetonitrile precipitation of proteins.

Due to the putatively heterogeneous nature of the compounds present in the hydro-alcoholic extract, a first separation of the different classes of compounds was performed by protein precipitation with acetonitrile organic solvent (ACN). ACN precipitation allows efficiently precipitation of abundant proteins (usually larger than ~40 kDa) in complex biological samples [94,95,96]. Due to its physicochemical properties, ACN is also widely used as solvent in the extraction of high value products; furthermore, salting-out as well as sugaring-out procedures were also used to trigger phase separation in ACN-water mixtures [97,98]. The natural presence of salts in the jellyfish samples allowed a spontaneous phase separation, yielding an upper layer that was primarily ACN (Upper Phase, UP in Figure 3), as described in Mathies and Austin [99], and a partitioning of the proteins in two fractions: Intermediate and Lower Phases (IP and LP), respectively (Figure 3). By this method, dilution of the specimen was avoided and cleaner solutions were available for analyses. Beside simplicity and rapidity, this procedure is found to be an effective method in complex sample protein separation [94,95,96] and it was used here for the first time for whole animal tissues. We found the phase separation method to be a very convenient approach, which allowed separating over the 90% of the proteins present in the extract. The phase equilibrium of ACN-water systems after the phase separation showed that the bottom phases (IP and LP) contain a certain amount of ACN, since they did not freeze at −20 °C [100]. This makes this phase separation method more attractive for bioactive molecules, since no strong organic solvent is used and no damage of freeze-sensitive proteins occurs in presence of the highly polar solvent ACN. We routinely used this method in our protocol before the samples were lyophilized and we propose this method for the extraction of bioactive molecules from marine gelatinous organisms.

### 2.3. Fraction Characterization

**Figure 4:**
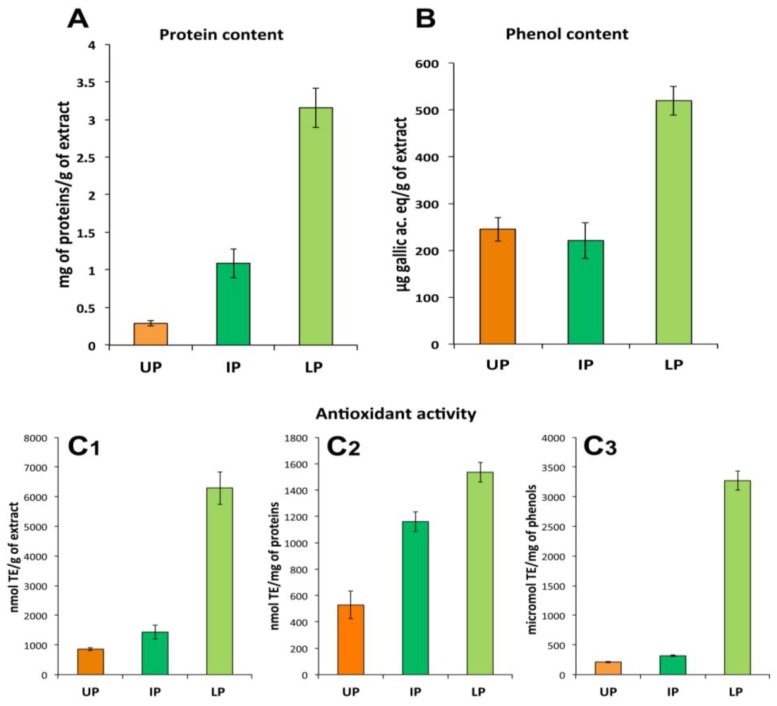
Protein (**A**) and total phenol (**B**) content and antioxidant activity (**C1**–**C3**) in the three fractions of the hydro alcoholic extract: UP, upper phase; IP, intermediate phase; LP, lower phase. Antioxidant activity data are expressed per gram of total extract (**C1**), per mg of proteins (**C2**) or phenols (**C3**) contained in each fraction. Data are mean ± SD of five independent experiments.

The UP consisted essentially of ACN-soluble compounds including non-polar or less-polar compounds. As expected, the IP and LP contained the most part of proteins (about 1.09 ± 0.19 mg and 3.29 ± 0.26 mg of proteins per gram of dried extract, respectively), where very low protein content was detectable in UP fraction (about 0.35 ± 0.03 mg/g of dry weight, DW, of extract). Phenol compounds were detected in all the three fractions, the most part being in the LP (about 520 μg of gallic ac. equivalents/g of dried extract) compared to UP and IP fractions (245 μg/g DW and 221 μg/g DW of extract, respectively). All the three fractions showed considerable antioxidant activity correspondent to 865, 1433 and 6294 nmol of Trolox equivalents (TE) per gram of dried sample in UP, IP and LP, respectively. The most antioxidant activity appeared related to the LP, since its value remained higher compared to the other fractions also when normalized to proteins or total phenols content (Figure 4C1–C3). Since the LP showed the highest content of both proteins and phenols, the nature of the antioxidant compound(s) cannot be identified at this level of analysis. 

**Figure 5:**
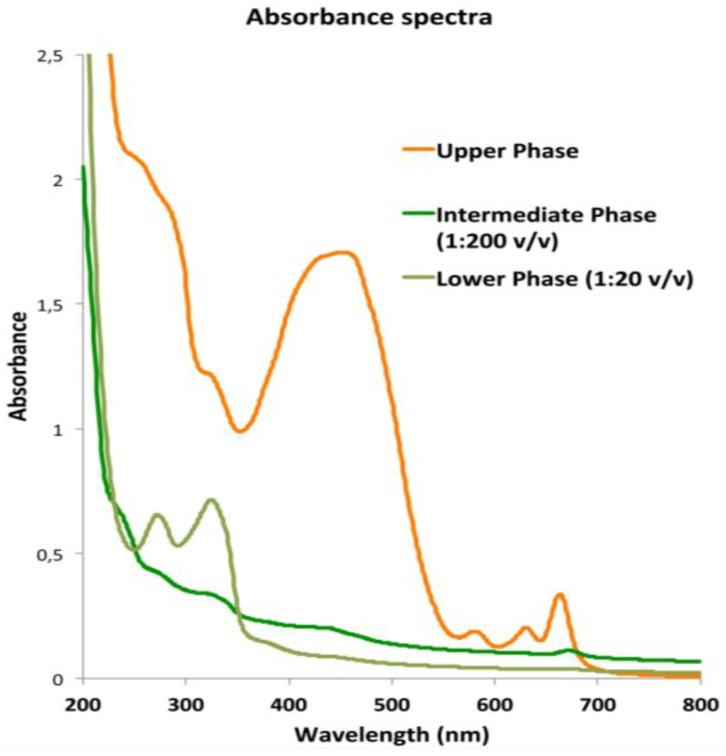
Graph of the absorbance spectra of upper phase (UP), intermediate phase (IP) and lower phase (LP) of the *C. tuberculata* extract measured from 200 to 800 nm in polar organic solutions. Blanks were with acetonitrile (UP) and water/acetonitrile (IP and LP).

The absorbance spectra of the three fractions measured in the visible and far-red region (Figure 5) showed that the absorption properties of the UP correspond to the characteristic absorption spectra of the photosynthetic pigments (chlorophylls and carotenoids) and pigment-protein-complexes recognized in polar organic solvent [45,101]. The fractions showed high absorbance from 215 to 280 nm where peptide bonds in proteins and free amino acids have maximum absorption. 

#### 2.3.1. Lipophilic Fraction: Upper Phase

**Figure 6:**
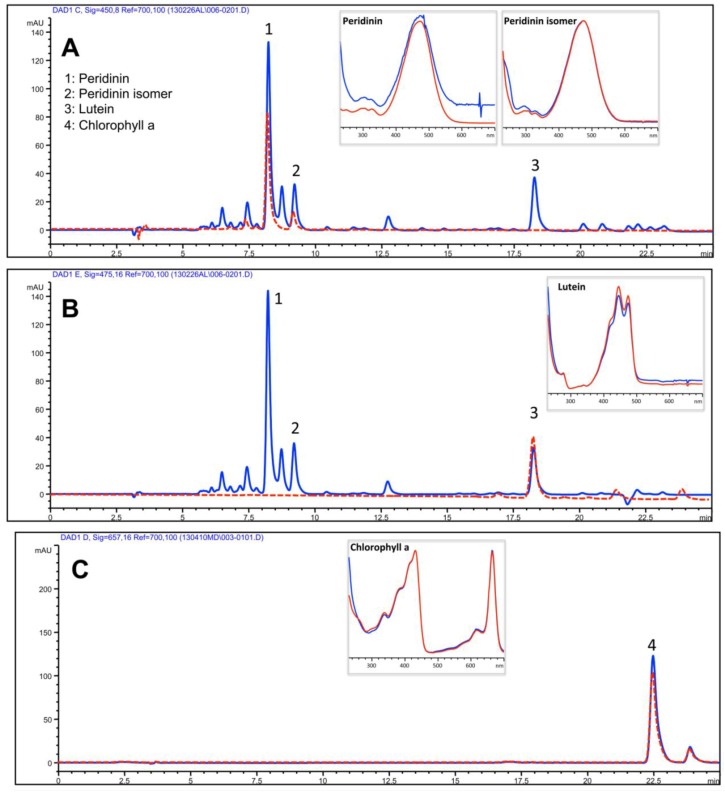
HPLC analysis of the isoprenoids in the UP. Absorbance was registered at 450 nm (**A**) and 475 nm (**B**) for carotenoids and at 657 nm (**C**) for chlorophylls. Peaks 1, 2, 3 and 4 correspond to peridinin, peridinin isomer, lutein and chlorophyll a, respectively, as confirmed by comparison with the retention time (dotted red line) and the absorbance spectra (boxes) of pure standard compounds. Red lines: standard compounds; blue lines: UP sample.

The yellowish to brown pigmented upper phase (UP) consisted essentially of ACN-soluble compounds, expectably including less polar and lipid components of both animal and dinoflagellate origin. A separation of isoprenoids was obtained using a reverse-phase C30 column and a mobile phase consisting of methanol, ammonium acetate aqueous solution/methanol (20/80 v/v) and terz-methyl butyl ether through an isocratic elution as described in the Experimental Section. The resulting HPLC profile of UP was dominated by the algal-derived chlorophyll a and xanthophylles: peridinin, peridinin isomer and lutein as showed by comparison with authentic standards (Figure 6A,B). Chlorophyll a was clearly detected in the UP fraction; isomers b and c were not detected in our experimental conditions (Figure 6C). The identification of compounds was carried out by means of authentic standards as described in the Experimental Section. 

The quantitative analysis (Table 1) showed that peridinin was the main xanthophyll detected and carotenoids were quantitatively predominant with respect to chlorophyll. 

**Table 1:** Isoprenoid composition in the upper phase (UP) of the 80% ethanol extract from *C. tuberculata*. Values are expressed as μg/g DW of total extract and are means ± SD of three independent experiments.

Isoprenoids	Upper Phase of hydro-alcoholic extract
	μg/g DW
Peridinin	385.1 ± 49.6
Peridinin isomer	186.5 ± 17.3
Lutein	108.7 ± 9.5
Chlorophyll a	20.3 ± 9.5

The presence of these photosynthetic pigments has been reported previously in HPLC studies of *Symbiodinium* [46]. The occurring light-harvesting pigment-protein complexes use chlorophyll to collect incoming photons, and most of these systems rely on carotenoids to supplement light-capture in the spectral region of maximal solar irradiance from 420 to 550 nm. Carotenoids, functioning as energy donors in antenna systems with an efficiency ranging from 30% to nearly 100%, also fulfil a crucial photoprotective role that preserves the structural and functional integrity of the photosynthetic apparatus. In endosymbiotic microalgae, the photoprotective role of photosynthetic pigments as well as other algal compounds [102,103] is likely exerted also for the benefit of the host jellyfish. 

**Figure 7:**
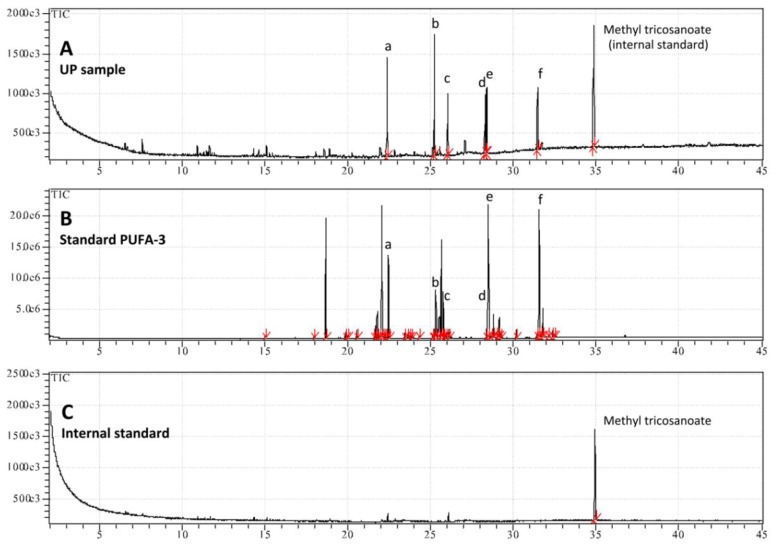
Patterns of GC-MS analysis of UP sample (**A**); standard PUFA No. 3 from menhaden oil (**B**); and methyl tricosanoate used as internal standard (**C**). The following fatty acid were identified after methyl esterification by comparing retention time with those of authentic standard (PUFA No. 3): (**a**) palmitic acid (C16:0), (**b**) *cis*-8,11,14,17-eicosatetraenoic acid C20:4 (ω-3), (**c**) Stearic acid (C18:0), (**d**) *cis*-5,8,11,14-eicosatetraenoic acid C20:4 (ω-6), (**e**) *cis*-5,8,11,14,17-eicosapentaenoic acid C20:5 (ω-3), (**f**) *cis*-4,7,10,13,16,19-docosahexaenoic acid C22:6 (ω-3).

In addition to the pigments, UP showed a significant lipid component and the amount of total fatty acids (FA) present was 2.450 ± 0.289 mg/g of dry weight of total extract. Fatty acids as methyl esters in the UP were determined separately using gas chromatography and verified by mass spectrometry (GC-MS). The GC-MS analysis revealed the presence of saturated (SFA) and polyunsaturated (PUFA) but not monounsaturated (MUFA) fatty acids (Figure 7). The hydroalcoholic extraction allowed a preferential removal of long chain polyunsaturated fatty acids; indeed the PUFA represented more than 70% of the total FA (Table 2). Mass spectral fragmentation (Figure S1) showed the presence of palmitic (C16:0) and stearic acids (C18:0) together with long chain polyunsaturated fatty acids belonging to ω-3 and ω-6 in the UP, including a considerable amount of the *cis*-8,11,14,17-eicosatetraenoic acid (ETA; C20:4, ω-3), and the occurrence of *cis*-5,8,11,14-eicosatetraenoic acid (AA; C20:4, ω-6), *cis*-5,8,11,14,17-eicosapentaenoic acid (EPA; C20:5, *n*-3), *cis*-4,7,10,13,16,19-docosahexaenoic acid methyl ester (DHA; C22:6, ω-3) (Figure 7 and Table 2).

The fatty acids belonging to ω-3 and ω-6 are essential diet components for animals, being involved in a number of biological processes including growth, development, tissue and cell homeostasis [104]. Several studies have reported biological activities of fatty acids from various microalgal species, like anti-bacterial [105,106,107] andanti-cancer activities [108]. Further characterization of the lipid content in the total jellyfish extract (TE) and its fractions is in progress.

**Table 2:** Fatty acids composition of the lipids present in the upper phase (UP) of the 80% ethanol extract from *C. tuberculata*. Values are expressed as percentages of total fatty acids and are means *±* SD of three independent experiments.

Fatty acids	Upper Phase of hydro-alcoholic extract
	% of total FA
**Saturated Fatty Acids (SFA)**	
Palmitic acid (C16:0)	18.9 ± 0.7
Stearic acid (C18:0)	9.5 ± 0.4
**Total SFA**	28.4
**Monounsaturated Fatty Acids (MUFA)**	n.d. ^1^
**Polyunsaturated Fatty Acids (PUFA)**	
*cis*-8,11,14,17-Eicosatetraenoic acid C20:4 (ω-3)	28.2 ± 2.6
*cis*-5,8,11,14-Eicosatetraenoic acid C20:4 (ω-6)	12.5 ± 0.4
*cis*-5,8,11,14,17-Eicosapentaenoic acid C20:5 (ω-3)	14.9 ± 1.4
*cis*-4,7,10,13,16,19-Docosahexaenoic acid methyl ester C22:6 (ω-3)	15.9 ± 0.9
**Total PUFA**	71.6
TOTAL (%)	100.0
Total (mg/g DW of the extract)	2.450 ± 0.289

^1^ n.d.: not detected.

In addition to pigments and lipids, UP contained also phenol compounds measured here as total phenols (Figure 4), whose complete characterization is in progress. 

#### 2.3.2. Protein Fractions: Intermediate and Lower Phases

**Figure 8:**
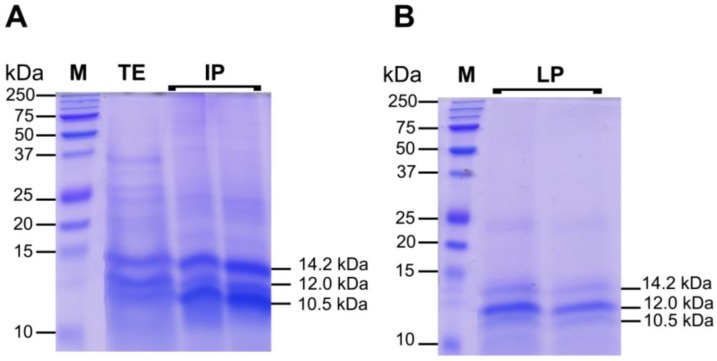
Polypeptide patterns, separated by 12% reducing SDS-PAGE, of 25 μg of total hydro-alcoholic extract before ACN precipitation (TE), and 25 μg of Intermediate phase (IF) (**A**), and Lower phase (LP) (**B**) of the extract after ACN precipitation. The molecular weight size marker (lane 1, M), in the range of 250–10 kDa, was run in parallel with samples for molecular weight estimation. Protein bands were visualized by staining gels with Coomassie R-250 dye.

The IP and LP were rich in proteins; IP contained also protein-pigment complexes where LP was pigment and lipid free (data not shown). To start the characterization of the proteinaceous components of *C. tuberculata* jellyfish extract, we separated the proteins present in TE, IP and LP by SDS-PAGE. The total jellyfish extract (TE) contained several proteins with various molecular sizes (Figure 8): Most polypeptides ranged from 10 to 50 kDa, but some bands over 50 kDa were also detected. After ACN precipitation, most proteins were included in IP and LP fractions, where a higher concentration of low molecular weight polypeptides was observed, as compared to the protein pattern in TE. As revealed by the SDS-PAGE separation (Figure 8A,B), three polypeptide bands at 14.2, 12.0 and 10.5 kDa appeared more neatly concentrated in the IP and LP fractions. 

Specifically, the IP fraction showed a higher concentration of the 10.5 and 14.2 kDa polypeptides, where LP seemed enriched in the 12.0 kDa polypeptide. Since a bright green color characterized both IP and LP extractswith a darker green former fraction (Figure 3B), it can be hypothesized that protein-pigment complexes from the symbiont zooxanthellae are found and differenly partitioned there. The peculiar chemical-physical features and their different affinity with ACN could be responsible of the polypeptide layering. The denaturating conditions of SDS-PAGE, breaking up quaternary protein structure and overcoming some forms of tertiary protein folding, induce degradation of protein-pigment complexes and differential polypeptide separations. 

Diverse peptides with a wide range of biological activities, including antioxidant, antimicrobial, antitumoral, and antiviral activities as well as toxins, have been discovered from different marine organisms [109,110,111,112]. Marine microalgae have been found to be a good source of proteins [113]. Protein content of *Navicula incerta* was found to be higher than lipid and carbohydrates, reaching a value over 50% of its dry matter [113,114] and many other microalgae species have showed similar protein content [115]. In addition, antioxidative peptides from marine algae are gaining attention as new antioxidative alternatives in the last few years [110,116,117]. Recently, a great deal of interest has been expressed in marine-derived bioactive peptides because of their numerous beneficial health effects such as antihypertensive, antioxidative, anticoagulant, and antimicrobial components in functional foods or nutraceuticals and pharmaceuticals due to their therapeutic potential in the treatment or prevention of disease. Presently, the commercial applications of microalgae are as nutritional supplements, natural dyes and skin care products [118], but there are few studies reporting the antioxidative or other activities of microalgae protein derived peptides [113,116]. 

The polypeptides in the ranges of 10–15 kDa and 20–40 kDa appeared to be the major protein components of our jellyfish total hydro-alcoholic extracts. These ranges of molecular weights are in agreement with the size of jellyfish venom [119,120], extracted from the cnidocytes of the scyphozoan jellyfish *Nemophilema nomurai* [9], *Rhopilema esculenta*, *Cynea nozakii*, *Aurelia aurita* and *Pelagia noctiluca* [14,15,121,122], thus we cannot exclude the occurrence of polypeptide *C. tuberculata* venom in our extract fractions. Whether the detected polypeptides are components of the jellyfish nematocysts, other jellyfish cell types, or derived from the symbiotic dinoflagellates, this remains to be investigated. Further study will be required to identify these molecular entities and polypeptide composition in the near future.

### 2.4. Biological Activities of Jellyfish Extract Fractions

#### 2.4.1. Effect on Cell Viability

**Figure 9:**
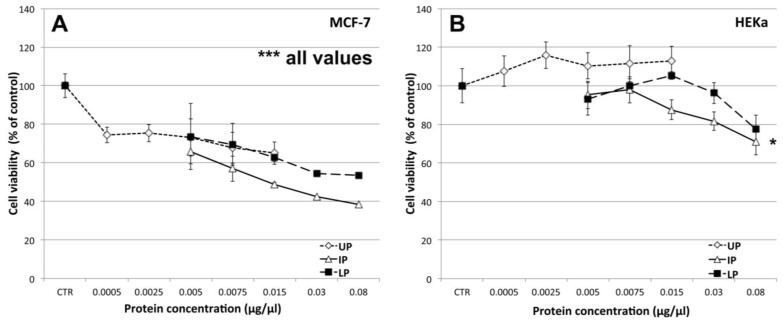
Effect of different concentrations of the three *C. tuberculata* extract fractions on the viability of MCF-7 (**A**) and HEKa (**B**) cells evaluated by the MTS assay. UP, Upper phase; IP, Intermediate Phase; LP, Lower Phase.Data are expressed as percentage of the control and represent the mean ± SD of three independent experiments (*n* = 12). * *p* ≤ 0.05; *** *p* ≤ 0.01. In **A**, *p* ≤ 0.01 for all values.

Different amounts of the three extract fractions were assayed for their effects on breast cancer (MCF-7) and epidermal keratinocyte (HEKa) human cell cultures (Figure 9). By the MTS assay method, we detected a highly significant (*p* ≤ 0.01) dose-dependent decrease of cell viability (as compared to the vehicle controls) of human breast cancer cells, at all tested concentrations, for each of the three jellyfish extract fractions (Figure 9A). The UP fraction was already effective at the lowest tested protein concentration (0.0005 μg/μL); at its highest tested concentration (0.015 μg/μL) the UP fraction was effective to reduce cancer cell viability by up to 65% of the control value. The two protein-enriched fractions, IP and LP, both induced a comparably remarkable and dose-dependent decline of MCF-7 cell viability. The IP showed the highest antiproliferative activity, resulting in a remarkable decrease of MCF-7 cell viability in a concentration dependent manner, by up to 38% of the control value when cell cultures were treated with 0.08 μg/μL of proteins. For the IP fraction, an exponential regression curve (*R*^2^ = 0.99813) best represents the inhibition curve for MCF-7, with the IC_50_ value coincident with the experimental treatment with 0.015 μg/μL of protein extract.

Surprisingly, no cytotoxic effect was observed in the non-malignant HEKa cells, when treated with the same concentrations of UP, IP and LP. No significant differences with the control values were found, except following treatment with the highest IP concentration (0.08 μg/μL, *p* < 0.05) (Figure 9B).

**Figure 10:**
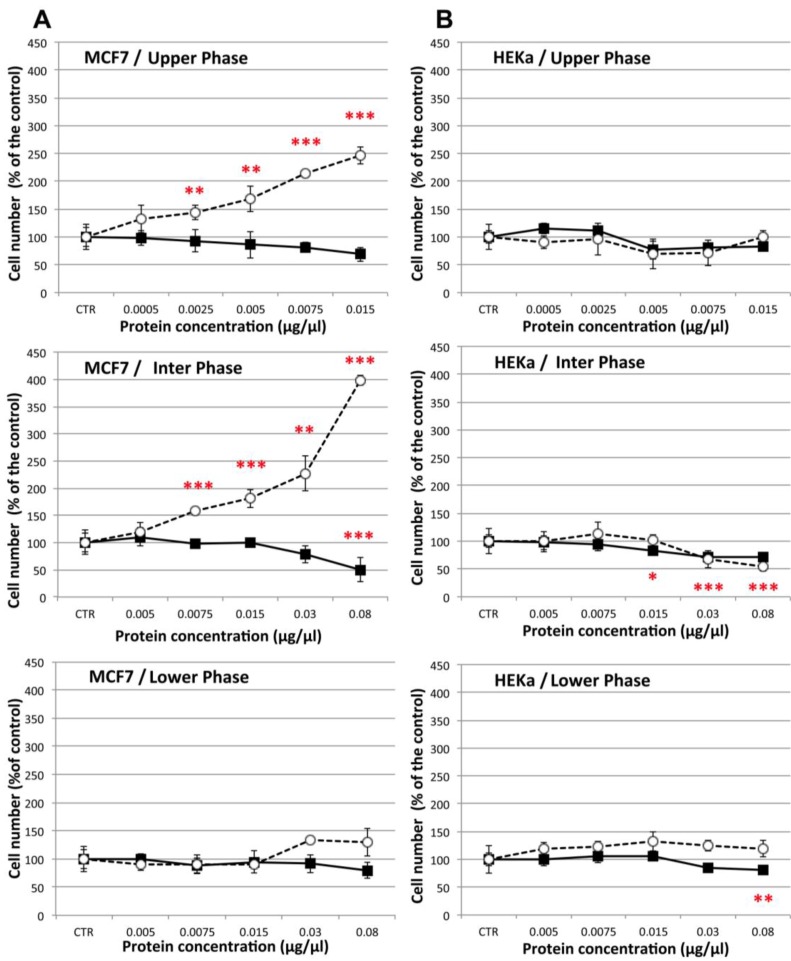
Effect of different concentrations of the three *C. tuberculata* extract fractions on the viability of MCF-7 (**A**) and HEKa (**B**) cells measured by Trypan blue dye exclusion associated to automated cell counting. Live (black squares) and dead (open circles) cells were calculated by automatic cell counting. Data are expressed as percentage of the control and represent the mean ± SD of three independent experiments (*n* = 9). * *p* ≤ 0.05; ** *p* ≤ 0.03; *** *p* ≤ 0.01.

To verify whether the cell viability decrease was due to cytotoxic/pro-apoptotic or cytostatic effect, the viability assays were also analyzed by Trypan blue dye exclusion associated to automatic cell counting, a method able to quantify live and dead cells (Figure 10). The results confirmed a significant (*p* ≤ 0.01) dose dependent decrease of cell viability in MCF-7 cancer cells treated with the highest doses of UP and IP (Figure 10A), and no significant differences in cell viability of non-malignant HEKa cells, as compared to the control, except for the highest tested concentrations of IP and LP (Figure 10B). At the same time, a remarkable dose-dependent increase of cell death in MCF-7 cancer cell cultures was recognizable after treatment with UP and IP at all tested concentrations.

Therefore, the antiproliferative effect of the *C. tuberculata* jellyfish extracts against breast cancer cells was clearly due to cytotoxic rather than cytostatic effects, at least during the 16 h incubation assay. On the contrary, the extract fractions seem to have no or weak cytostatic effect upon non-malignant HEKa cells. Indeed no significant differences in the dead cell number, as compared with the control, were recorded in HEKa treated with the three extract fractions, and only IP and LP affected the HEKa cells through a slight decrease of live cell number.

The effect on MCF-7 breast cancer cell was higher by treatment with the UP and IP fractions of *C. tuberculata* extract, whose protein contents were lower. Thus, although the dose-response was related to the protein content, the involvement of other classes of compounds, like phytochemicals from the symbiotic algae cannot be excluded. 

The apparent absence of cell-specific (MCF-7 *vs.* HEKa) effects of LP fractions (Figure 10A,B, bottom graphs) might be related to the potential concentration of venom-like substances in this proteinaceous fraction. Antiproliferative activity has been reportedfor jellyfish venoms. Recently, thevenom of the mauve stinger *P. noctiluca* was suggested to have moderate cytotoxic effects on U87 cancer cells and to promote apoptotic death of non-malignant Vero cells by generation of intracellular ROS [15,123,124]. However in these studies, appropriate controls with protein denatured treatments were missing. More generally, only a small fraction of the vast diversity of cnidarian toxins has been studied in detail [125] and comparative studies on their toxicity on cancer and non-cancer cells are still lacking. To our knowledge, our results show for the first time a cell-specific cytotoxic activity against MCF-7 breast cancer cells induced by components of *C. tuberculata* jellyfish extracts, an activity which does not manifest against non-malignant HEKa cells in the same experimental conditions. 

#### 2.4.2. Effect on Cell-Cell Communication

**Figure 11:**
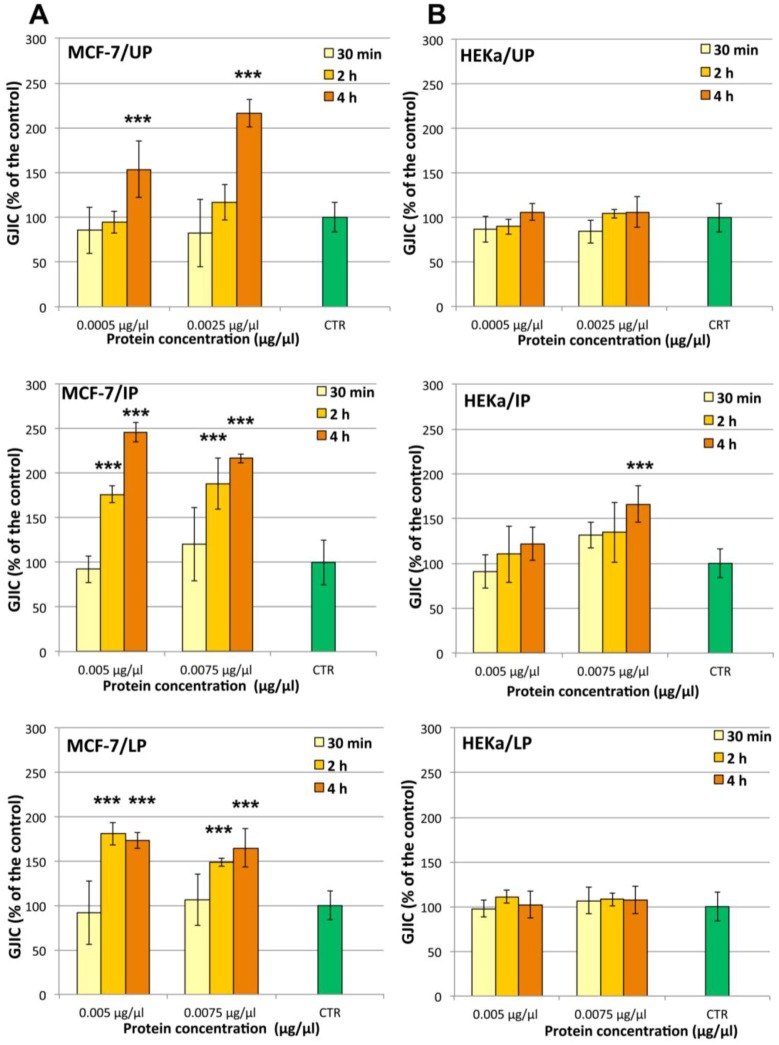
Dose- and time-response treatments with the three *Cotylorhiza* extract fractions on the gap junction intercellular communication (GJIC) in MCF-7 (**A**); and HEKa cell cultures (**B**). UP, Upper phase; IP, Intermediate Phase; LP, Lower Phase.Data are expressed as percentage of the relative control and represent the mean ± SD of three independent experiments (*n* = 6). *** *p* ≤ 0.01.

In order to identify the action mechanism(s) of the observed cell-specific antiproliferative activity, the influence of *C. tuberculata* extract on the gap junction intercellular communication (GJIC) functionality was studied. Non-cytotoxic amounts of UP, IP and LP for HEKa cells, which were shown to induce antiproliferative effect on MCF-7, were used to challenge the two different cell populations for 30 min, 2 h and 4 h (Figure 11). The results obtained by cytotoxicity assays by MTS or direct cell counting were strongly correlated with the observed changes in GJIC functionality of both MCF-7 and HEKa cell types during the first 2–4 h of exposure to the jellyfish extract fractions. 

A remarkable time- and dose-dependent enhancement of GJIC was evident in MCF-7 cells treated with all the three fractions of the jellyfish extract (Figure 11A). Indeed, a 4 h treatment with both UP concentrations (0.0005 and 0.0025 μg/μL of protein) resulted into increases of GJIC functionality by up to 150 and 220% of the control value, respectively. A stronger effect was evident for both IP and LP, which affected the GJIC functionality with both tested concentrations (0.005 and 0.0075 μg/μL) at 2 h and 4 h of treatment but not after 30 min. After 2 h of treatment, IP was the most active fraction, inducing a GJIC increase two and a half times higher than the control.

The effect on GJIC in non-malignant cells HEKa was much less detectable. All treatments on HEKa cells were carried out with non-cytotoxic doses of UP, IP and LP. No significant changes in GJIC in HEKa was recorded after treatment with all fractions at all checked concentrations and times, except for a 4 h treatment with IP at 0.0075 μg/μL, producing an increase of GJIC functionality of about one and half times (Figure 11B). 

The ability to enhance GJ mediated cell-cell communication seems to be peculiar of *C. tuberculata* extract fractions on both considered cell types. In addition, the GJIC enhancement would appear not related to transient effects on GJ proteins, such as phosphorylation state changes, since no significant changes in the GJIC functionality were observed after 30 min of treatment with all the three fractions at all checked concentrations.

The correlation between the effect on GJIC and cell viability enable us to hypothesize that the GJIC enhancement during the first h of the treatment, in MCF-7 breast cancer cells underlies the cell-specific cytotoxicity. It is known that a transient increasing of GJIC functionality is able to trigger apoptosis and to determine the cell death leading to reduction in cancer cells [68]. Since the MCF-7 cells are a model for chemotherapy-induced apoptosis as well as chemo-resistance studies [126], we believe that the evidenced up-regulation of GJIC could be considered one of the mechanisms able to reduce the tumorigenic phenotype. 

At our knowledge no data on GJIC modulation by bioactive marine compounds have been so far reported. The only available data derive from an *in silico* study addressing the efficiency of interactions between some naturally occurring marine compounds with active site residues of target proteins [81]. The analysis was carried out using marine compounds from marine algae, sponges, corals, bacteria, cnidarians, and marine fish targeted against gap junction proteins (Connexin 26, Connexin 43) and cell adhesive molecules (cadherins, integrins) via molecular docking studies. Several compounds from Cnidaria, as well as marine algae, were demonstrated to potentially bind connexin proteins [81].

Many compounds able to modulate GJIC have anticancer effects and most of which are phytochemicals [70]. The UP of the *C. tuberculata* extract contains mainly carotenoids and photosynthetic pigments. Many carotenoids were demonstrated to exert antitumor promoter activity [71,127,128], including xanthophills as astaxanthin and canthaxanthin, which are common in marine organisms. These compounds proved to actively enhance cell-cell communication by direct connexin over expression [70,129]. As the UP jellyfish fraction actively affected both GJIC functionality and cell viability in MCF-7 cells, the involvement of carotenoids or similar phytocompounds should be considered.

Here we showed that PUFAs were peculiar components of the *C. tuberculata* extract. A relationship between GJIC functionality and PUFA has been also observed. Modulation of cardiac connexins by *n*-3 PUFAs probably contributes to the antiarrhythmic effects of oral treatment withfish oils in dog model [130]. Several finding have demonstrated the modulation of connexin expression and distribution by PUFAs [130,131,132]. The mechanism probably relies on some non-specific role of PUFAs as components of biologic membranes. PUFA-rich, phospholipid-cholesterol-poor microdomains provide the molecular platform for the permanent reconstruction of the membrane and adaptor protein complexes of most cell junctions as TJ, GJ and AJ [133]. Indeed, *n*-3 PUFAs (e.g., EPA and DHA) form a part of cell membrane, replacing other mostly unsaturated fatty acids upon incorporation, and thereby modulating membrane fluidity and cellular functions [134,135,136]. DHA enrichment of membrane phospholipids was shown to increase gap junction coupling capacity in cultured astrocytes [131]. The enhanced gap junction coupling of DHA-enriched cells was associated with a more functional distribution of connexin 43 at cell interfaces and more of the main phosphorylated isoform of connexin 43. It seems therefore very likely that incorporated *n*-3 PUFA can modulate Cx43 channel (as well as other channel) function, via affecting microdomains of cell membrane through which the channel protein penetrates [136].

Our experimental finding cannot exclude the possible involvement of jellyfish venom compounds in the observed effects on human cells. Although envenomation effects, including cytolysis and ion channel perturbations [137], could be related with mechanisms involving gap junction functions, to date there is no evidence of direct effects of nematocyst venoms on GJIC functionality and connexins. The increase in membrane conductance evoked in *Xenopus oocytes* by incubation with the *Cassiopea* *xamachana* venom did not correspond to opening of Cx38 hemi-gap-junction channels [138].

## 3. Experimental Section

### 3.1. Chemicals and Media

Methanol, ethanol, hexane, ammonium acetate, terz-methyl butyl ether, acetonitrile (HPLC grade) and acetic acid were purchased from Merck (Darmstadt, Germany); potassium persulfate (dipotassium peroxdisulfate) and 6-hydroxy-2,5,7,8-tetramethylchroman-2-carboxylic acid (Trolox) were purchased from Hoffman-La Roche. 2,20-Azinobis (3-ethylben-zothiazoline-6-sulfonic acid) diammonium salt (ABTS), gallic acid, Folin-Ciocalteu’s phenol reagent, FAME mix (C_8_–C_24_), PUFA No. 3 (from menhaden oil), boron trifluoride (BF_3_), Trypsin-EDTA solution and methyl tricosanoate were purchased from Sigma-Aldrich. MTS CellTiter 96^®^ AQueous Non-Radioactive Cell Proliferation Assay (Promega, Madison, WI, USA), RPMI 1640 medium, Dulbecco’s phosphate buffered saline (PBS), l-glutamine (GLN), antibiotic-antimycotic stabilized solution (AA), heat inactivated fetal bovine serum (FBS), Trypan blue solution 0.4%, Epilife Medium, HKGS, Penicillin-Streptomycin solution and Lucifer Yellow CH dilithium salt, were purchased from Life Technologies (Carlsbad, CA, USA). Acrylamide solution was purchased from Euroclone (Pero, Milano, Italy). Peridinin, lutein in ethanol and chlorophylls in acetone were purchased from DHI Water & Environment (Copenhagen, Denmark). All other reagents were of analytical grade. 

### 3.2. Jellyfish Samples

Live jellyfish specimens of *C. tuberculata* were collected in south Adriatic and Ionian Sea (Otranto and Santa Caterina, Lecce, Italy) in September 2010 and 2011. After biometric measurement (weight and diameter), samples were immediately frozen in liquid nitrogen and stored at −80 °C until lyophilization. Large specimens were cut and quarter radial sectors of the jellyfish were used as homogeneous portion representative of the whole animal. Frozen jellyfish were freeze-dried for 4 days at −55 °C in a chamber pressure of 0.110 mbar in a freeze dryer (Freezone 4.5L Dry System, Labconco Co. Thermo Scientific, Kansas City, MO, USA). After lyophilization, the dry weight was recorded and the samples were stored at −20 °C until use.

### 3.3. Preparation and Fractionation of the Jellyfish Extract

Four grams of lyophilized jellyfish sample were subjected to hydro alcoholic extraction by stirring in 16 volumes (w/v) of 80% ethanol, overnight at 4 °C. Samples were then centrifuged at 9000× *g* for 30 min, at 4 °C, and the supernatants indicated as Total Extract (TE) were used for biochemical assays. TE was concentrated by vacuum rotary evaporator (Buchi), at low temperature and protected from light, in order to evaporate the ethanol, and then frozen in liquid nitrogen and lyophilized. 

Dried extract was weighed and 50 mg were solubilized in 1 mL of acetonitrile/water 1:1 (v/v) at 4 °C, in order to separate proteins. After gentle shacking, the suspension was left on ice in order to facilitate the phase separation. Three phase were obtained (Figure 2) indicated as Upper Phase (UP), Intermediate Phase (IP) and Lower Phase (LP). Protein concentration, antioxidant activity and total phenol content were evaluated in the TE and in UP, IP, and LP fractions. 

### 3.4. Protein Concentration

Protein content in TE, UP, IP and LP was estimated by modified Bradford assay [139] using bovine serum albumin (BSA) as a standard for curve construction.

### 3.5. Determination of Phenol Content

The total content of phenols was determined by a modified Folin-Ciocalteau colorimetric method. The test solutions containing 100 μL of sample were mixed with 500 μL of Folin-Ciocalteu’s phenol reagent and with 500 μL of 7.5% sodium carbonate (Na_2_CO_3_). After 2 h, at room temperature in the dark, the absorbance was spectrophotometrically measured at 760 nm. The calibration curve was plotted *versus* concentrations of gallic acid ranging from 25 to 200 μg/mL, used as standard. The results were expressed as gallic acid equivalents per gram of dry extract.

### 3.6. *In Vitro* Antioxidant Activity Analysis

The total antioxidant activity was determined spectrophotometrically by using the Trolox Equivalent Antioxidant Capacity (TEAC) method, as described in Leone *et al.* [71], using the radical cation ABTS^•+^ and Trolox as standard. Ten microliters of the jellyfish extract or fractions were assayed into 1 mL of the reaction mixture and the absorbance decrease was measured at 734 nm. Solutions of 80% ethanol, acetonitrile or acetonitrile/water were used as control. A Trolox calibration curve in a range of 2.5–20 μM was prepared under the same conditions of the samples. The antioxidant capacity of the samples was calculated, on the basis of the inhibition exerted by standard Trolox concentrations at 734 nm, inhibition time being fixed at 6 min. Results were expressed as nmol of Trolox equivalents per gram of sample or per mg of contained proteins. 

### 3.7. Fatty Acid Profiles Determination

Fatty acid methyl esters (FAME) were obtained using BF_3_ according to Szczesna-Antczak *et al.* [140] with some modifications. Upper phase (1 mL) was saponified at 90 °C for 20 min with 0.5 M KOH in methanol (3 mL). Forty-nine micrograms of the internal standard (methyl tricosanoate) were added before saponification. The fatty acids were methylated by adding 14% BF3 in MeOH (2 mL) and heating at 90 °C for 10 min. After cooling, hexane (1 mL) was added and vigorously stirred for 30 s before the addition of 1 mL of sodium chloride solution (0.6%). The esterified sample was left at 4 °C for better phase separation. After collecting the supernatant, another 1.0 mL of hexane was added and stirred. The supernatant was collected and added to the previous fraction. The sample was concentrated to a final volume of 1.0 mL for GC-MS analysis.

### 3.8. HPLC Analysis of the UP Fraction

The pigments in upper phase were separated according to Guaratini *et al.* [141] using an Agilent 1100 HPLC. Analyses were carried out as described by Fraser *et al.* [142], with slight modifications. Isoprenoids were separated using a reverse-phase C30 column (5 μm, 250 × 4.6 mm) (YMC Inc., Wilmington, NC, USA) with mobile phases consisting of methanol (A), 0.2% ammonium acetate aqueous solution/methanol (20/80 v/v) (B), and terz-methyl butyl ether (C). The isocratic elution was as follows: 0 min, 95% A and 5% B; 0 to 12 min, 80% A, 5% B, and 15% C; 12 to 42 min, 30% A, 5% B, and 65% C; 42 to 60 min, 30% A, 5% B, and 65% C; 60 to 62 min, 95% A, and 5% B. The column was re-equilibrated for 10 min between runs. The flow rate was 1.0 mL/min, and the column temperature was maintained at 25 °C. The injection volume was 10 μL. Absorbance was registered by diode array at wavelengths of 450 and 475 nm for carotenoids and 657 nm for chlorophylls. Isoprenoids were identified by comparing their retention times and UV-visible spectra to authentic standards peridinin, lutein in ethanol and chlorophylls. 

### 3.9. GC-MS Analysis

The analyses were performed on a GC-MS system, Shimadzu GC-17A version 3.0 (Shimadzu Co., Kyoto, Japan), with MS QP5050A according to Talà *et al.* [143]. Compounds were separated on DB-5 capillary column having 30 m length, 0.25 mm ID and 0.25 μm thickness. The GC parameters were as follows: the column temperature was 80 °C at the injection then programmed at 10 °C/min to 150 °C, at 5 °C/min to 250 °C and maintained at 250 °C for 15 min. Split injection was conducted with a split ratio of 50:1, the flow-rate was 1.0 mL/min, carrier gas used was 99.999% pure helium, the injector temperature was 250 °C and the column inlet pressure was 74 kPa. The MS detection conditions were as follows: 250 °C interface temperature; ionization mode, EI^+^; electron energy, 70 eV; scanning method of acquisition, ranging from 30 to 450, for mass/charge (*m*/*z*) optimization. Spectrum data were collected at 0.5 s intervals. Solvent cut time was set at 2 min and 45 min retention time enough for all fatty acids separation all. Compounds were identified by using online NIST-library spectra and published MS data. Moreover, FAME mix (C_8_–C_24_) and PUFA No. 3 (from menhaden oil) authentic standards were used to confirm MS data. 

### 3.10. Protein Analysis by SDS-PAGE

Different component proteins in the total extract and fractions (TE, IP and LP) and their molecular weights were assessed by electrophoresis (SDS-PAGE) on 12% separating gel with a 4% stacking gel. The Precision Plus Protein Dual Color Standard (Bio-Rad, Hertfordshire, UK) was used as molecular weight marker. The protein bands were stained with 0.25% Coomassie brilliant blue R250 in 10% acetic acid and 50% methanol for 20 min. The gel was then detained with 10% acetic acid and 30% methanol overnight.

### 3.11. Cell Cultures

Breast cancer cell line, MCF-7, containing the estrogenic receptor, was obtained from the European Collection of Cell Cultures (ECACC, London, UK). Cell line was routinely grown in RPMI-1640 medium supplemented with 10% FBS, 2 mM glutamine (GLN), 50 U/mL penicillin G, 50 μg/mL streptomycin (all from GIBCO) in 75 cm^2^ plastic flasks (Corning, NY, USA) at 37 °C in a 5% CO_2_ humidified atmosphere. Cells were passaged at 70%–80% confluence, about twice a week by trypsinization.

HEKa cells (Invitrogen) were cultured in Epilife Medium supplemented with HKGS, 50 U/mL of penicillin G and 50 μg/mL of streptomycin (all from GIBCO). Cells were plated at density of 150,000 cell/mL in 75 cm^2^ for HEKa and 250,000 cell/mL for MCF7 plastic flasks and incubated at 37 °C in a 5% CO_2_ humidified atmosphere and trypsinized when reached 80%–90% confluences.

### 3.12. Cell Viability and Treatments

Cell viability was assayed by MTS assay, as indirect measure of viable cell number, and by Trypan blue dye exclusion associated to automated cell counting (Countess^®^ Automated Cell Counter, Invitrogen Carlsbad, CA, USA), as suitable method to asses the real number of live and dead cells, in order to overcoming the possible inaccuracy of MTS assay in presence of supplements or new bioactive chemicals [144,145].

#### 3.12.1. MTS Assay

MCF-7 cells were seeded in flat bottomed 96-well plates (Corning, NY, USA) at 25 × 10^4^ cells/well in 200 μL of RPMI medium supplemented with FBS, GLN and AA and HEKa were seeded at 25 × 10^4^ cells/well in 200 μL supplemented Epilife Medium, and allowed to attach for 24 h at 37 °C in a 5% CO_2_ humidified atmosphere. Subsequently, the culture medium was replaced with complete medium containing the extract fractions at the following protein concentrations: UP, ranging from 0.0005 to 0.015 μg/μL and IP and LP from 0.005 to 0.08 μg/μL. For negative controls, the test compounds were replaced with solvent only (0.1% acetonitrile) or medium only, controls were included on each plate. Each treatment was performed in quadruplicate. The cells were then incubated for 16 h at 37 °C and 5% CO_2_ and assayed for vitality by MTS assay. Twenty microliters of CellTiter 96^®^ Aqueous One Solution Reagent (Promega, Madison, WI, USA) were added to each well according to the manufacturer’s instructions. After 1 h in culture the cell viability was determined as assessment of metabolic activity by measuring the absorbance at 490 nm using a 3550 Ultra Microplate Reader (Biorad Instruments) (Bio-Rad, Hertfordshire, UK). The assay was performed in quadruplicate for three independent experiments and the results were expressed as a percentage of control.

#### 3.12.2. Trypan Blue Dye Exclusion Assay

Cell viability was determined by Trypan blue (Invitrogen™, Carlsbad, CA, USA) dye exclusion in order to evaluate the cytotoxic effect induced by extract treatment. HEKa cells were seeded at a density of 6 × 10^5^ cells and MCF-7 were seeded at 10^6^ cells in 35 mm plate in 2 mL of the relative complete medium and treatments were performed when cells reached the 90% of confluence. Treatments and controls were performed as for MTS assay. At the end of treatment, cultures were trypsinized and a sample of cell suspension (20 μL) was mixed with Trypan blue (20 μL; 0.4% dye solution). A Cell Countess system (Invitrogen) was used to count the number of viable and non-viable cells and the percentage viability was calculated. Three independent experiments were performed in triplicate for each treatment and controls with vehicles and medium only.

### 3.13. Estimation of GJIC by Scrape-Loading Dye Transfer (SL/DT) Assay

GJIC was assessed using the SL/DT technique described by El-Fouly [146]. Cells were seeded at 6 × 10^5^ cells/plate (HEKa) and at 10^6^ cells/plate (MCF-7) in 35 mm cell culture dishes, and incubated at 37 °C and 5% humidified atmosphere. Only cells at 95% confluence were used for the experiments herein, they were incubated with the test compounds (UP, IP and LP fractions) at the two lower tested concentrations in the vitality tests. The incubation with test substances was 30 min, 2 h or 4 h. Controls with only medium or vehicles were included in each experiment. After treatment, the MCF-7 and HEKa cells were rinsed carefully with PBS without Ca^2+^ Mg^2+^, and then scraped and incubated with 0.5 mL of 0.1% (w/v) Lucifer Yellow CH (Molecular Probes, Invitrogen), for 3 min. The cells were then washed with PBS and fixed with 4% paraformaldehyde. The distance that Lucifer Yellow had travelled through gap junctions was observed with a laser scanning confocal microscope (Carl Zeiss, Munchen, Germany) and the number of fluorescent cells (cell in communication via gap junctions) was compared with the controls. Six random images were recorded for each plate and means were considered. Three independent experiments for each cell type were performed in triplicate for each treatment and for controls with the vehicles and medium only.

### 3.14. Laser Scanning Confocal Microscopy

Fresh jellyfish tissues containing symbiont cells were imaged using a Zeiss LSM Pascal confocal microscope excited using a 488 nm (argon) and 543 nm (He–Ne) lasers. Emissions were collected using two detection channels within 505–530 nm (virtual green) and over 560 nm (virtual red) emission. Following spectral mapping of innate cell fluorescence, online profiling was used to image a consistent number of clustered cells in tissues from different jellyfish anatomical portions. The offline tools of confocal microscopy were used to measure the cell and subcellular dimensions.

### 3.15. Statistical Analysis

Statistical analysis was performed using a Student’s *t*-test and an analysis of variance (ANOVA) test to analyse any differences in the measured endpoints observed between controls and the treatment groups. In the GJIC assays, these tests were used to analyse fluorescent cell average values. The mean and standard deviation (SD) were calculated for all data.

## 4. Conclusions

This study revealed that partially characterized fractions of an extract of the zooxanthellate *Cotylorhiza tuberculata* jellyfish exert antioxidant ability and a cell-specific cytotoxicity against MCF-7 breast cancer cells rather than against non-malignant HEKa cells. The cytotoxicity correlated with an effective enhancement of cell-cell communication mediated by gap junction communication, which may represent one of the targets in the action mechanism of the extract bioactivity. Since the goal for an effective anticancer drug is to prove a cytotoxic selective effect preferentially expressed for cancer cells rather non-malignant cells, we believe the jellyfish *C. tuberculata* deserves further studies to verify the effects of the extract fractions on additional cell lines. Further studies directed towards the isolation of the active compound(s) and in-depth knowledge on antiproliferative activity and the molecular targets of marine natural compounds may provide valuable information for the development of therapeutic approaches towards new strategies of cancer prevention and control. In this framework, on going work in our laboratory will verify the hypothesis of the role of *C. tuberculata* extracts as transcriptional activators of connexin proteins.

In several Mediterranean coastal areas, *C. tuberculata* populations seasonally reach high biomass values. Therefore, our results suggest increased attention should be paid to the jellyfish-algal symbiont consortium of *C. tuberculata* for its potential as a source of bioactive compounds of nutraceutical and chemopreventive relevance. The exploitation of the jellyfish/algal consortium, occurring with large unexploited biomasses in coastal areas, should be enhanced by promoting new studies towards the complete characterization of the bioactive molecules and their molecular action mechanisms.

More generally, jellyfish biomasses represent a source of pharmacological research largely unexplored and may lead to a future different perception of jellyfish outbreaks in our seas.

## Supplementary Files

Supplementary File 1 Supplementary Information (PDF, 439 KB)
