# Supplementary material for: Extract from the Zooxanthellate Jellyfish Cotylorhiza tuberculata Modulates Gap Junction Intercellular Communication in Human Cell Cultures
Source: Mar Drugs. 2013 May 22;11(5):1728–62. doi: 10.3390/md11051728 (PMC3707171; doi:10.3390/md11051728)
Supplement: Supplementary File 1 — Supplementary Information (PDF, 439 KB) [file marinedrugs-11-01728-s001.pdf]

Supplementary Information

Table S1. Biometric measures and dry weight of *Cotylorhiza tuberculata* specimens caught in the summer 2011.

Specimens	Umbrella diameter (cm)	Total fresh weight (g)	Fresh weight/ diameter ratio	Dry weight (g)	Dry weight (% of FW)
**C-11-002**	6.3	19.2	3.05	0.91	4.7
**C-11-003**	7.4	32.0	4.32	1.91	6.0
**C-11–007**	7.5	30.3	4.04	1.69	5.6
**C-11–005**	8.0	39.4	4.93	2.14	5.4
**C-11–008**	8.3	38.3	4.61	2.10	5.5
**C-11–004**	9.3	52.5	5.64	2.86	5.5
**C-11–006**	16.0	428.4	26.78	97.25	22.7
**C-11–009**	17.5	491.4	28.08	159.21	32.4
**C-11–011**	18.0	389.5	21.64	40.61	8.3
**C-11–001**	19.0	489.3	25.75	104.78	26.9
**C-11–010**	25.5	1080.0	42.35	212.76	19.8
**C-11–012**	25.5	1179.0	46.24	409.11	34.7
**C-11-013**	29.0	1770.0	61.04	507.99	28.7

Table S2. Fresh and dry weights of *Cotylorhiza tuberculata* specimens and of the lyophilized hydro-alcoholic extract. Data are referred to some representative extractions.

Specimen	Total lyophilized jellyfish (TE)		Lyophilized hydro-alcoholic extract		
	Dry weight	Percentage of fresh weight	Dry weight	Percentage of TE	Percentage of fresh weight
	(g)	(% of FW)	(g)	(% of DW)	(% of FW)
#1	31.91	22.7	13.16	41.3	9.4
#2	65.91	32.4	26.68	40.5	13.1
#3	71.23	26.9	30.79	43.2	11.6
#4	49.04	25.8	24.28	49.5	12.8
Mean ± SD *		27.0 ± 4.0	23.72 ± 7.5	43.6 ± 4.1	11.7 ± 1.7

* Data are means ± SD.

Figure S1. Mass spectra fragmentation patterns of the UP sample (**A**) and the standard PUFA No. 3 (from menhaden oil) (**B**). The following fatty acids were identified in the UP sample after methyl esterification and comparison with the mass fragmentation spectra of the standards (PUFA No. 3): (**a**) palmitic acid (C16:0), (**b**) *cis*-8,11,14,17-eicosatetraenoic acid C20:4 (ω-3), (**c**) Stearic acid (C18:0), (**d**) *cis*-5,8,11,14-eicosatetraenoic acid C20:4 (ω-6), (**e**) *cis*-5,8,11,14,17-eicosapentaenoic acid C20:5 (ω-3), (**f**) *cis*-4,7,10,13,16,19-docosahexaenoic acid C22:6 (ω-3).

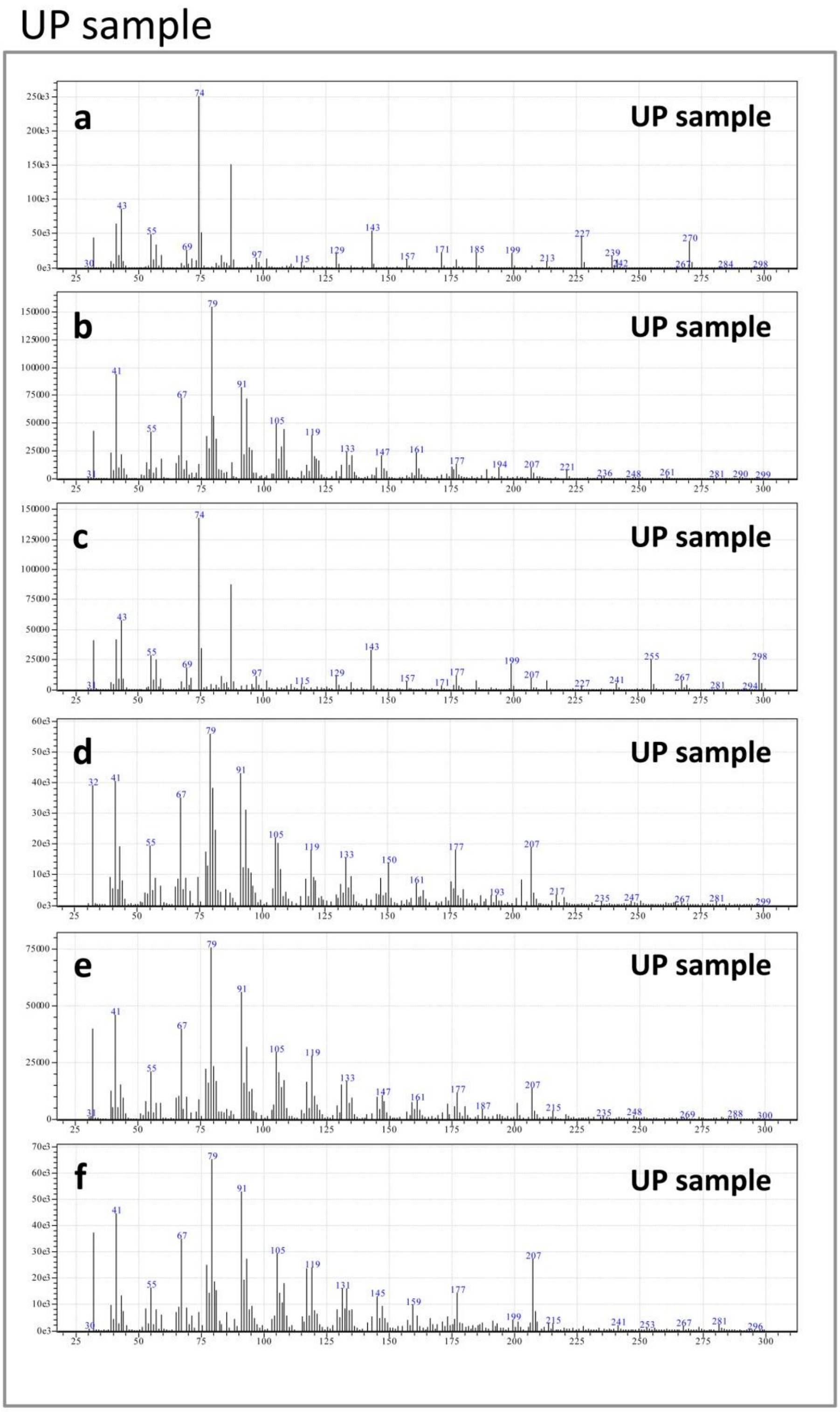

(**A**)

Figure S1. *Cont.*

Standard PUFA Mix No. 3

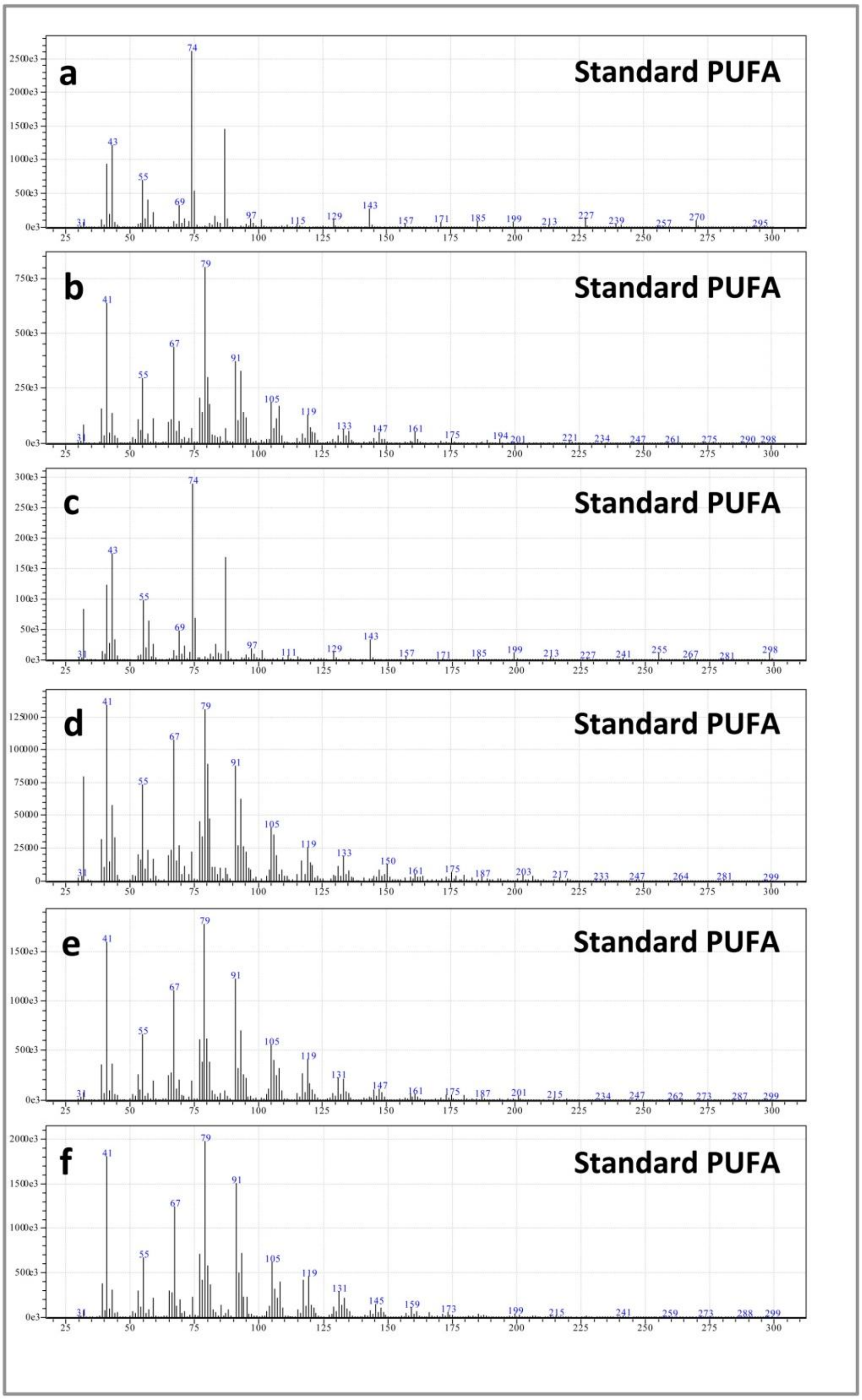

(**B**)
